# Development of a potent neutralizing nanobody against canine distemper virus hemagglutinin protein

**DOI:** 10.3389/fimmu.2025.1585793

**Published:** 2025-05-08

**Authors:** Lirong Xiao, Zhuqing Wu, Jingliang Su, Qingmin Wu

**Affiliations:** National Key Laboratory of Veterinary Public Health Security, College of Veterinary Medicine, China Agricultural University, Beijing, China

**Keywords:** canine distemper virus, hemagglutinin protein, nanobody, phage display, neutralizing antibody

## Abstract

Canine distemper virus (CDV) is the etiological agent of canine distemper. The virus can infect canids irrespective of age, sex, or breed, leading to a highly contagious and lethal disease that seriously threatens the health of canids, fur animals, and wildlife. Although vaccination can currently prevent CDV infection, developing effective emergency treatment drugs remains crucial. Nanobodies derived from camelid or shark heavy chain-only antibodies can effectively inhibit viral infections, suggesting their potential as therapeutic agents for treating CDV infection. In this study, we utilized a phage display nanobody library constructed from immunized alpacas and isolated a nanobody (Nb-6C6) that specifically binds to the CDV hemagglutinin (H) protein. Nb-6C6 was successfully expressed in mammalian cells and exhibited high binding affinity to CDV H (EC_50_ = 0.174 µg/mL). Neutralization assays further revealed that Nb-6C6 could effectively neutralize CDV (IC_50_ = 1.773 µg/mL). Fusion of Nb-6C6 with canine IgG Fc resulted in homodimers, significantly increasing its neutralizing activity by up to 4.6-fold. AlphaFold3 analysis indicated that the neutralizing capacity of Nb-6C6 against CDV is attributed to an interaction between residue D106 in the CDR3 region and the conserved residue R408 of the H protein. These findings suggest that the nanobody Nb-6C6 and its bivalent form exhibit high-affinity binding and potent neutralizing activity against CDV, highlighting their potential as promising therapeutic candidates for the treatment of CDV infection.

## 1 Introduction

Canine distemper virus (CDV) is an enveloped RNA virus that belongs to the genus *Morbillivirus* within the Paramyxoviridae family (1). CDV infection can cause a highly contagious and lethal disease in Canidae known as canine distemper (CD), a crucial infectious disease that threatens the health of dogs and other species and has severely affected the development of dog breeding and economic animal husbandry. This disease is clinically characterized by diphasic fever, leukopenia, gastrointestinal and respiratory catarrh, and frequent pneumonic and neurological manifestations (2–4). CDV is a promiscuous virus that can infect many different host species and has a worldwide distribution. Members of the families Procyonidae, Mustelidae, Felidae, and Viverridae are recognized as susceptible. The expansion of the host range has often been reported, with sporadic spillover of the virus into endangered wildlife, such as panda populations and African wild dogs, posing significant threats to wildlife conservation programs (5, 6). Moreover, owing to its remarkable ability to cross species barriers, there have been instances of CDV infecting primates, raising concerns regarding the potential zoonotic risk of CDV (7, 8). Live attenuated CDV vaccines have significantly reduced infection rates in domestic dogs across mainland China. However, cases of CD can still be observed in animal hospitals because of inappropriate vaccination (9, 10) or genetic variations in field CDV strains that antigenic shifts (11). The continuous mutation of CDV, the emergence of new genotypes, and changes in the ecological environment often compromise the effectiveness of vaccines. In clinical practice, symptomatic and supportive treatments frequently prove ineffective. By contrast, infected dogs have shown positive responses to antibody therapy, highlighting the potential of neutralizing antibody-based treatments for CDV infection. Monoclonal antibodies (mAbs) have been approved for diagnosing and treating CDV in dogs in China. This advancement provides a promising strategy for the emergency treatment of CDV infections.

CDV is a single-stranded, negative-sense RNA virus with a genome structure that includes six transcription units (N-P-M-F-H-L) organized in a linear form (12). The hemagglutinin (H) protein, a type II transmembrane glycoprotein on the surface of the CDV envelope, assembles into a tetrameric structure by forming a dimer-of-dimers, which is decisive in determining host specificity (13). Like other members of the Paramyxoviridae family, the H glycoprotein can utilize signaling lymphocyte activation molecule (SLAM) and nectin cell adhesion molecule 4 (Nectin-4) as specific entry receptors to facilitate the attachment of the virus to the host cell membrane (14–17). A conformational change in the F protein subsequently initiates the fusion of the viral and plasma membranes (18). Recent studies have identified low-density lipoprotein receptor-related protein 6 (LRP6) as an alternative host receptor that promotes CDV Onderstepoort vaccine strain infectivity, expanding our understanding of viral entry mechanisms (19). Additionally, the CDV H is the antigenic determinant site that elicits specific cytotoxic T lymphocyte responses in the host. This protein can induce the production of a large quantity of neutralizing antibodies and is key for the development of new vaccines and therapeutic formulations (20–22). Consequently, the development of specific therapeutic agents based on the H protein is highly important for preventing and controlling CDV.

Currently, most commercially available mAbs against CDV are produced by mouse hybridoma technology (23). However, because of the immunogenicity of antibodies, the repeated use of murine mAbs can cause adverse reactions in dogs, such as allergic responses, which may compromise their therapeutic efficacy (24). The variable domains of heavy chain-only antibodies (VHHs), also known as nanobodies, are monomeric antigen-binding domains derived from camelid or shark heavy chain-only antibodies (25, 26). Nanobodies have been investigated as important therapeutic alternatives for combating viral infections because of their excellent binding affinity and stability. Additionally, they can be more efficiently and effectively expressed in bacteria, yeast, and fungi. These advantages imply that, as therapeutic antibodies, nanobodies can potentially reduce production and transportation costs in future development. Currently, a bivalent VHH fusion protein, caplacizumab (Cablivi), has been approved by the US Food and Drug Administration for the treatment of adult-acquired thrombotic thrombocytopenic purpura, indicating the druggability of nanobodies and their fusion proteins (27–29). Compared with the antibody fragments of most animals, nanobodies have a small molecular weight (approximately 15 kDa), enabling them to recognize hidden epitopes within antigens that are inaccessible to conventional antibodies, which is advantageous for use as drugs and diagnostic reagents (30–33). Compared with conventional antibodies, nanobodies exhibit excellent stability, good solubility, and high specificity and are easy to engineer into multivalent nanobodies, bispecific antibodies, and nanobody drug conjugates (34). The small size of nanobodies results in a short circulation time *in vivo*, potentially limiting their therapeutic efficacy (35). However, structural optimization strategies, particularly Fc fusion technology, can effectively extend nanobody half-life (36). This approach not only enhances antibody affinity but also activates Fc-fragment-mediated antibody effector functions, including antibody-dependent cellular cytotoxicity (ADCC) and antibody-dependent cellular phagocytosis (ADCP) (37, 38). While significant progress has been made in applying antibodies to treat human diseases, nanobody research in veterinary medicine remains in its early stages.

Here, we constructed a phage display nanobody library by immunizing alpacas with a live attenuated CDV vaccine and screened the library to identify high-affinity CDV H-targeting nanobodies with desirable neutralization activities. We constructed a bivalent fusion protein with canine IgG Fc to form a divalent structure to increase nanobody affinity for the CDV H and its neutralizing activity against CDV. We subsequently modeled the structure of the CDV H-nanobody (Nb) complex to predict the molecular interactions between the nanobodies and the CDV H and determine the key site of interaction exerting antiviral effects.

## 2 Materials and methods

### 2.1 Cells, vectors, and virus

Vero E6 and HEK293T cells were cultured in Dulbecco’s modified Eagle’s medium (DMEM, Thermo Fisher Scientific) supplemented with 10% fetal bovine serum (FBS, Gibco), 100 U/mL penicillin and 100 µg/mL streptomycin, and by incubation at 37°C under an atmosphere of 5% CO_2_. The CDV strain Onderstepoort (GenBank accession number AF378705.1) was propagated and preserved in our laboratory in Vero E6 cells. HEK293T cells were used for recombinant nanobody expression. For prokaryotic expression of the CDV H and nanobodies, we employed pET-32a and pET-28a vectors (Novagen). A pcDNA3.4 vector (Novagen) was used for mammalian cell expression of nanobodies and Nb-Fc. The short phagemid vector, M13K07 helper phage, and *Escherichia coli* SU320 and SU5alphaF′ strains, purchased from Chengdu Ablink Biotechnology, were stored in our laboratory for nanobody library construction.

### 2.2 Expression, purification, and identification of recombinant CDV H

Total RNA was extracted from Vero E6 cells infected with CDV vaccine strain Onderstepoort using TRIzol reagent (Invitrogen, USA) to produce the recombinant CDV H. The truncated H gene (59**–**604 aa) was amplified using GoScript Reverse Transcription System (Promega) with the primer pairs CDV-H-F (5′-GCCATGGCTGATATCGGATCCCGATTTCACCAAGTATCAACTAGCA-3′) and CDV-H-R (5′-TTGTCGACGGAGCTCGAATTCACGGTTACATGAGAATCTTATACGGA-3′) according to the manufacturer’s instructions. The PCR product was purified and ligated into the pET-32a vector (Novagen) with verification by Sanger sequencing. A polyhistidine tag was added to the 5′ end of the fragment to facilitate the detection and purification of the recombinant protein. Then, the pET32a-H recombinant plasmid was transformed into *E. coli* BL21 (DE3) competent cells. Recombinant H expression was induced by adding isopropyl-β-d-thiogalactopyranoside (IPTG). Bacterial cells were subsequently centrifuged and lysed by sonication, and the recombinant protein was purified using Ni-NTA Beads 6FF Agarose (Solarbio) following the manufacturer’s protocol. The protein was further verified by SDS-PAGE and western blotting.

### 2.3 Phage display library construction

**Figure 1:**
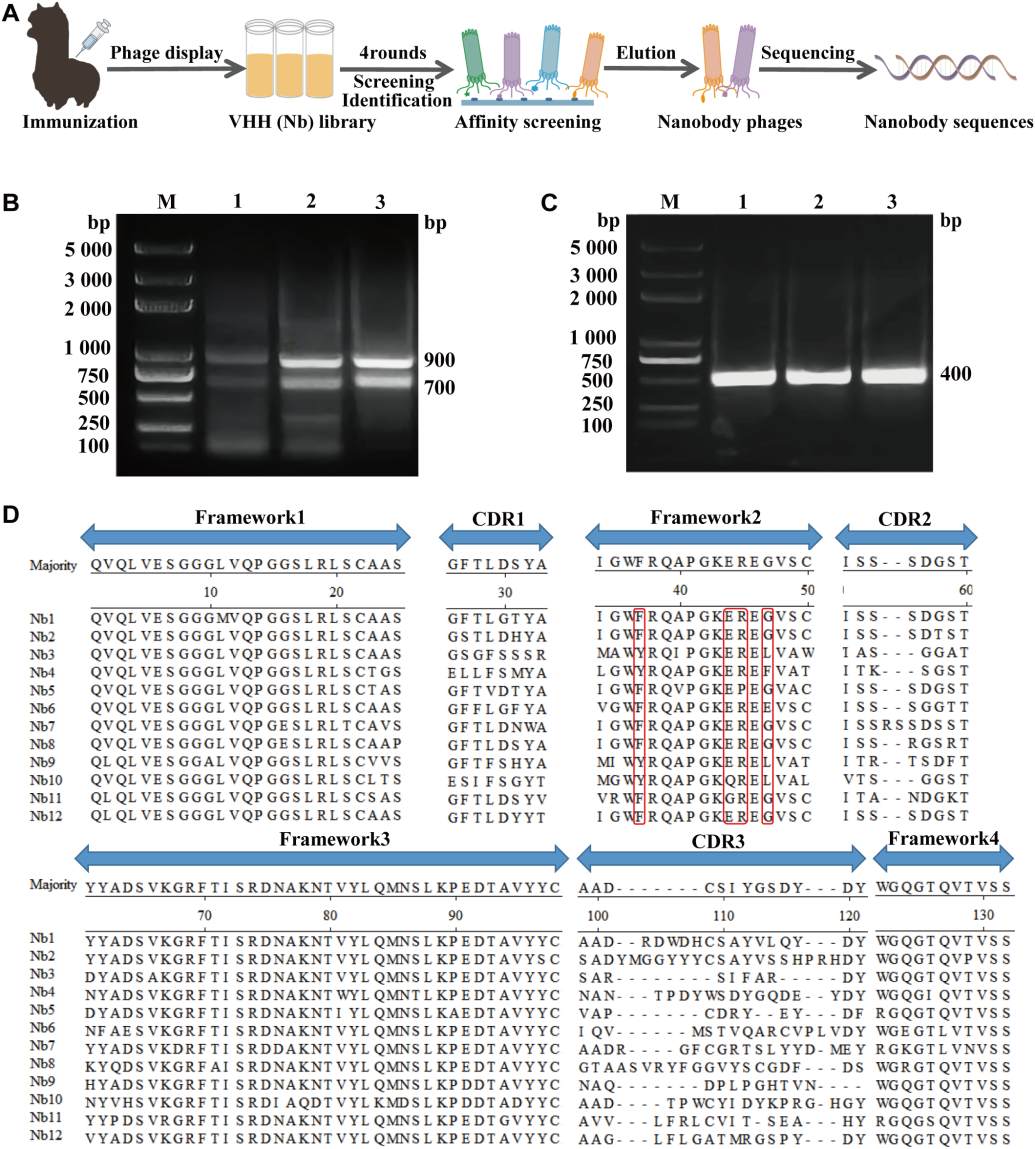
Nanobodies library construction against CDV. **(A)** Schematic representation of screening nanobodies from the immunized alpaca using the phage display platform. **(B)** The first round of PCR resulting in an approximately 700 bp fragment. **(C)** The second round of PCR resulting in an approximately 400 bp fragment. **(D)** Alignment of the amino acid sequences of 12 randomly selected nanobodies based on CDRs.

A healthy 2-year-old alpaca (*Vicugna pacos*) was immunized four times at 2-week intervals with live attenuated CDV Onderstepoort vaccine (Nobivac) through bilateral cervical lymph node multipoint injections to construct a phage display nanobody library, following established protocols (39). Serum samples were collected before the first immunization and 5 days after the final boost. The antibody titer against CDV H in the serum was measured by indirect enzyme-linked immunosorbent assay (ELISA) using recombinant CDV H as the coating antigen. Subsequently, 50 mL of blood was collected in a vacuum tube one week after the final immunization to prepare lymphocytes. The nanobody (VHH) library was constructed according to the procedures presented in **Figure 1A**. Total RNA was extracted from 10^7^ peripheral blood lymphocytes using TRIzol reagent (Invitrogen) and reverse-transcribed into cDNA with reverse transcriptase (Promega). The VHH genes (approximately 400 bp) were amplified by nested PCR with primer pairs CALL001/CALL002 and AlpVHH-F/R1/R2 (**Supplementary Table S1**). Then, the nested PCR products were inserted into the linearized phagemid vector. Recombinant phagemids were transformed into electroporation-competent *E. coli* SU320 cells, which were cultured on 2YT agar plates supplemented with glucose and carbenicillin overnight. The positive rate of the nanobody library was tested with primer pairs Short-F/R (**Supplementary Table S1**) by PCR amplification. Clones were scraped from the plates and stored at −80°C in 2YT with 20% glycerol.

### 2.4 Screening and identification of CDV H-specific nanobodies

To screen for nanobodies that specifically interact with CDV H, we utilized the phage antibody library constructed in the previous step for screening. We performed four rounds of screening on 10 µg of immobilized CDV H in 4 microtiter plate wells as described previously (40). We used four uncoated wells as negative controls in each round. Nonspecific binding sites on the wells were blocked with a 2% bovine serum albumin (BSA) blocking buffer (Solarbio, China). Approximately 4 × 10^11^ recombinant phage particles were added to uncoated wells, incubated at room temperature (RT) for 1 h, then transferred to coated wells and incubated for 2 h. Nonspecifically bound phages were removed by washing ten times with phosphate-buffered saline (PBS) + 0.05% Tween 20 (PBST). After washing, the retained phages were eluted and amplified in *E. coli* SU5alphaF′ cells, infected with M13K07 helper phages, and subsequently purified using PEG 8,000/NaCl precipitation for the next round of selection. After four rounds of screening, 576 randomly selected clones from the third and fourth round plates were cultured in liquid medium and induced with 1 mM IPTG to express nanobodies. The supernatants were tested by the indirect ELISA with an anti-M13-horseradish peroxidase (HRP) antibody conjugate (Sino Biological) to detect CDV H-specific nanobodies. Finally, all positive clones were sequenced and grouped by their complementary determining region sequences.

### 2.5 Expression of CDV H-nanobodies by *E. coli*

To characterize the functions of the selected nanobodies, they were first expressed and purified in *E. coli*. To obtain nanobodies against CDV H, six VHH genes encoding 1H7, 6G1, 5E12, 1H12, 2E8, and 6C6 were successfully ligated into the pET-28a vectors (Novagen, USA) and transfected into *E. coli* BL21 (DE3) cell lines to express high-yield nanobodies and assess their expression. First, the full-length sequences of selected nanobodies were amplified by PCR using 28a-Nbs-F/R primer pairs (**Supplementary Table S1**). They were subsequently cloned and inserted into the *Bam*HI/*Xho*I site of pET28a with Flag and His tags at the C-terminus. The positive plasmids were sequenced and analyzed using MegAlign software to confirm their successful construction. The recombinant positive plasmids were subsequently transformed into *E. coli* BL21 (DE3) chemically competent cells to induce expression at 37°C for 8 h by adding 0.5 mM IPTG. The expressed nanobodies were purified with Ni-NTA Beads 6FF Agarose (Solarbio) and eluted with 500 mM imidazole. Affinity-purified nanobodies were dialyzed against PBS to eliminate the imidazole. SDS-PAGE was subsequently used to analyze the expression and purification of nanobodies.

### 2.6 Expression of CDV H-nanobodies in HEK293T cells

To ensure proper protein folding, the nanobodies were expressed in HEK293T cells. Codon-optimized nanobody sequences were synthesized and cloned into the pUC57 cloning vector (Tsingke Biotech). The optimized nanobody genes were amplified by PCR with primer pairs pcDNA3.4-Nbs-F/R and pcDNA3.4-F1/R1 (**Supplementary Table S1**). Then, the PCR products were subcloned into the pcDNA3.4 eukaryotic expression vector, fused with an N-terminal signal peptide and a C-terminal 3×Flag tag followed by a His tag. Subsequently, the recombinant plasmids were transfected into HEK293T cells with polyethylenimine (PEI, Sigma-Aldrich, 408727). The nanobodies were harvested from the cell culture supernatants 72 h post-transfection, and the cells were fixed for indirect immunofluorescence detection. His-tagged nanobodies were purified on a Ni-NTA Beads 6FF Agarose column. The expression of the antibodies was verified by indirect immunofluorescence and western blotting. Finally, the purified nanobodies were separated on a 12% SDS-PAGE and stained with Coomassie blue to confirm purity, followed by functional characterization to evaluate biological activity.

### 2.7 Nanobody binding ability evaluation by ELISA

ELISA was performed to detect the binding activity of the obtained nanobodies with CDV H. In brief, a Maxisorp 96-well immune plate (42592, Costar) was coated overnight at 4°C with 0.1 µg of CDV H antigen. The coated plates were blocked with 2% BSA in PBST at 37°C for 2 h. Serially diluted nanobody solutions (containing a 3×Flag or Fc tag at the C-terminus) were added to the plates, which were incubated with the solutions for 1 h at 37°C. After five washes with PBST, bound nanobodies were detected with HRP-conjugated DYKDDDDK-tag monoclonal antibody (1:10,000, Proteintech) or HRP-conjugated rabbit anti-dog IgG Fc secondary antibody (1:10,000, Jackson ImmunoResearch Laboratories). After washing, 100 µL 3,3′,5,5′-tetramethylbenzidine (TMB, Solarbio) per well was added and reacted in the dark for 15 min; the reaction was stopped by adding 50 µL of 2M H_2_SO_4_. Finally, the absorbance at 450 nm was measured with a Synergy H1 plate reader (Biotek), and the data were analyzed using GraphPad Prism 8.0 software.

### 2.8 Immunofluorescence assay and western blotting

To identify nanobodies expressed in HEK293T cells, transfected cells were fixed with prechilled acetone/methanol (1:1) at RT for 20 min and then incubated with CoraLite Plus 488-conjugated DYKDDDDK monoclonal antibody (1:500, Proteintech). To verify the binding of nanobodies to CDV, the Vero E6 cells were inoculated with 100 tissue culture infective dose 50% (TCID_50_) CDV when the cells reached 80% confluence in a 24-well plate. After incubation for 1 h at 37°C under an atmosphere of 5% CO_2_, the cells were maintained in DMEM supplemented with 1% FBS. In parallel, a cell culture well was maintained as a negative control. After 72 h of incubation, infected cells were fixed using the procedure described above. Nonspecific binding sites on the cells were blocked using immunofluorescence blocking buffer, followed by three washes with PBS. The Vero E6 cells were incubated with nanobody expression supernatants at 37°C for 1 h, followed by adding CoraLite Plus 488-conjugated DYKDDDDK-tag monoclonal antibody. After washing, cell nuclei were stained with the blue-fluorescent DNA dye 4′,6-diamidino-2-phenylindole (DAPI) (Solarbio) for 10 min at RT. Mouse monoclonal antibody against CDV nucleoprotein (1:300, Santa Cruz Biotechnology) was used as a positive control, and DyLight 488 AffiniPure goat anti-mouse IgG (1:500, EarthOx) was used as the secondary antibody. Finally, the stained cells were observed directly using fluorescence microscopy (Olympus Corporation, Japan).

For western blotting, protein samples were separated by 12% denaturing SDS-PAGE and then transferred to a polyvinylidene fluoride membrane. Membranes with bound proteins were incubated in a blocking buffer (5% skim milk in PBST) at 4°C overnight and then washed five times with PBST. HRP-conjugated DYKDDDDK-tag monoclonal antibody (diluted 1:10,000) or HRP-conjugated rabbit anti-dog IgG Fc secondary antibody (diluted 1:10,000) was used to detect antibody expression. To verify the binding of nanobodies to CDV H, nanobodies were used as the primary antibody, and HRP-conjugated DYKDDDDK-tag monoclonal antibody was used as the secondary antibody. After incubation at RT for 1 h, the membrane was washed, and the signal was then developed using a chemiluminescence substrate (ECL reagent; Cwbiotech). The ChemiDoc imaging system (Bio-Rad) was used for stain-free gel visualization.

### 2.9 CDV neutralization assay

Neutralization assays were performed using HEK293T-derived nanobodies to maintain structural and functional integrity. The neutralizing capacity against CDV Onderstepoort strain was then evaluated by IFA. In brief, 2-fold serial dilutions of Nb-6C6 or 6C6-Fc were incubated with the same volume of 200 TCID_50_ CDV at 37°C for 1 h. Then, the mixtures were added to Vero E6 cells in 96-well plates (Corning) for 1 h at 37°C. After removing the mixtures, 100 µL of DMEM with 1% FBS was added to each well. The cells were incubated in a 5% CO_2_ incubator at 37°C for 72 h and then fixed as previously described. After that, we performed IFA by using a mouse monoclonal antibody against the nucleoprotein of CDV (1:300) as the primary antibody and DyLight 488 AffiniPure goat anti-mouse IgG (1:500) as the secondary antibody to assess the neutralizing ability of the antibodies.

To assess the neutralizing ability of the antibodies more precisely, we further performed a standard focus reduction neutralization test (FRNT). The antibodies and viruses were diluted and incubated as previously described. Then, the 200 µL mixtures were added to Vero E6 cells in 12-well plates in triplicate and incubated at 37°C for 1 h. After removing the mixture, 1 mL of 1.6% methylcellulose 4000 cP (Sigma-Aldrich) was added to each well. The plates were incubated in a CO_2_ incubator at 37°C for 3 days and then fixed with a prechilled acetone/methanol (1:1) mixture for 3 h at RT. After washing with PBS, nonspecific binding sites on the cells were blocked with a blocking buffer at RT for 2 h. Subsequently, the cells were stained with a mouse monoclonal antibody against the nucleoprotein of CDV (1:300) and HRP-conjugated goat anti-mouse IgG secondary antibody (1:10,000, Sino Biological). The foci were visualized using TMB One Solution (Yeasen). The half-maximal inhibitory concentration (IC_50_) values were derived from nonlinear regression analysis of focus counts using a four-parameter logistic regression and analyzed using GraphPad Prism 8.0 software.

### 2.10 Construction and characterization of 6C6-Fc

To explore the potential of Nb-6C6-based antiviral treatments and improve its neutralization activity further, we fused the canine IgG Fc region to the C-terminus of Nb-6C6 to construct canine heavy chain antibodies (**Supplementary Table S1** lists the primers pcDNA3.4-6C6-Fc-F1/R1, pcDNA3.4-6C6-Fc-F2/R2, and pcDNA3.4-F2/R2 that were utilized). The sequences of canine IgG Fc (CH2 and CH3 domains) and the hinge region are available in the GenBank database (accession number AAL35301.1). Transfect the eukaryotic recombinant plasmid pcDNA3.4-6C6-Fc into HEK293T cells for expression. We also expressed a separate Fc region to be used as a control in subsequent experiments. The two proteins were purified using protein A-Sepharose affinity chromatography medium and analyzed under reducing and nonreducing conditions by western blotting with an HRP-conjugated rabbit anti-dog IgG Fc secondary antibody. Furthermore, we used indirect ELISA to evaluate the binding of 6C6-Fc to the CDV H. Finally, we also evaluated the neutralizing activity of 6C6-Fc against CDV through IFA and FRNT.

### 2.11 Structural modeling and interaction analysis of CDV H and Nb-6C6

The 3D homology models of protein structures were predicted using AlphaFold3 (https://www.alphafoldserver.com). Molecular docking was then employed to analyze the interactions between CDV H and Nb-6C6 and define the structural basis underlying the neutralization mediated by Nb-6C6. The predicted binding sites were further analyzed using the PDBePISA (https://www.ebi.ac.uk/pdbe/pisa/), which also provides thermodynamic parameters of the complexes, including the binding free energy (Δ^i^G, in kcal/mol) and the interface area (Å^2^). Finally, the 3D structures of the Nb-6C6 and CDV H binding models were visualized using PyMol software (Schrödinger).

### 2.12 Statistical analysis

Statistical analyses were performed using GraphPad Prism version 8.0 software. Data are presented as mean ± SD, derived from three independent experiments. The statistical differences were evaluated by Student’s t test for the two groups. Comparisons among multiple groups were performed using a two-way ANOVA test with Bonferroni post-test. P-values < 0.05 were considered statistically significant (*P < 0.05; **P < 0.01; ***P < 0.001).

## 3 Results

### 3.1 Expression and purification of recombinant CDV H protein in *E. coli*

**Figure 2:**
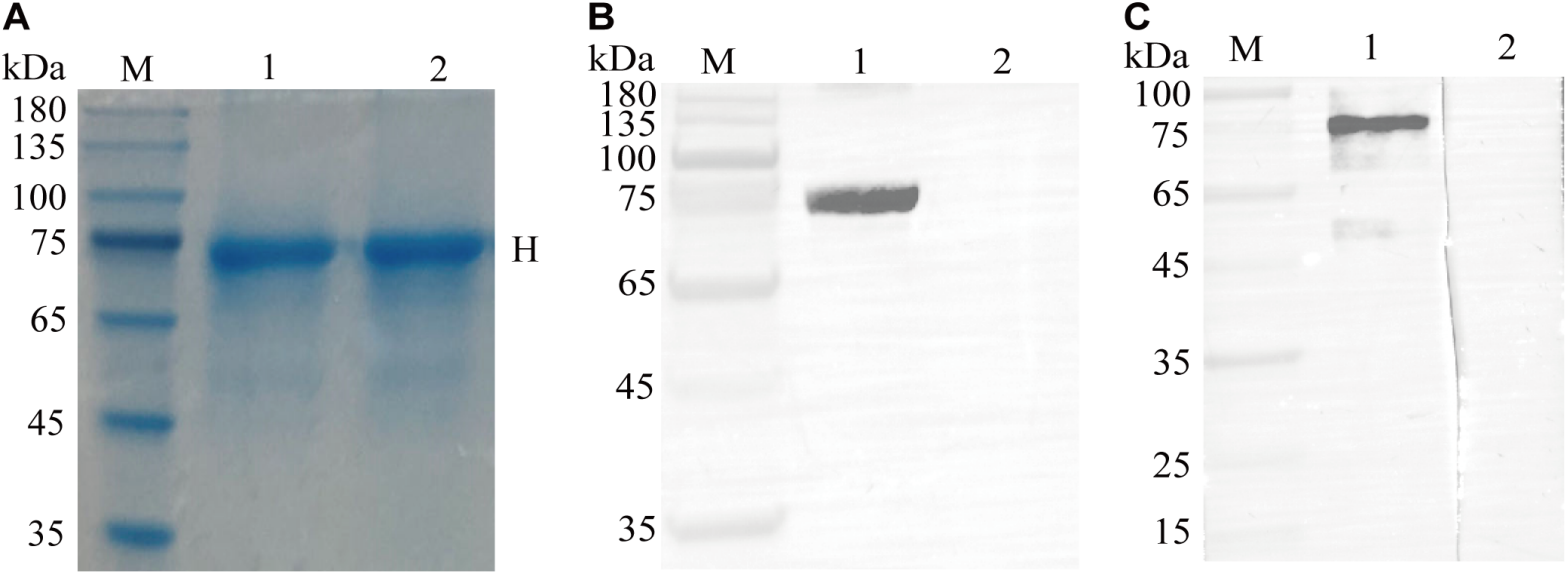
Expression, purification, and identification of the recombinant CDV H protein. **(A)** SDS-PAGE analysis of recombinant CDV H protein expressed in *E coli*. M: M5 Prestained Protein Marker; lanes 1-2: purified protein. **(B)** Western blotting identified the recombinant protein with a monoclonal antibody against the His tag. Lane 1: purified recombinant protein; Lane 2: pET-32a vector control. **(C)** Antigenic analysis of western blotting. Lanes 1-2: same as **(A)**, reaction with the immunized and un-immunized alpaca sera, respectively.

Using *E. coli* BL21 (DE3) cells for expression, SDS-PAGE revealed that the CDV H had the expected molecular size of approximately 75 kDa, and highly purified recombinant protein was obtained (**Figure 2A**). Western blotting revealed that the recombinant protein reacted with the HRP-conjugated His tag monoclonal antibody (**Figure 2B**). Furthermore, the recombinant protein was recognized by the serum from the immunized alpaca (**Figure 2C**). These results suggested that the CDV H protein was correctly expressed in *E. coli*.

### 3.2 Construction of a phage display nanobody library

After the last vaccine immunization, the antibody level against the CDV H protein in alpaca serum was tested using ELISA. Using the recombinant CDV H as the coating antigen, antibodies were detected at a high titer of 1:64,000 (**Supplementary Figure S1**). Then, we successfully amplified the VHH genes (approximately 400 bp) using nested PCR (**Figures 1B, C**). After ligation and transformation, a phage display nanobody library comprising approximately 2.1 × 10^7^ individual clones was successfully constructed. We selected 24 clones from the phage display nanobody library. PCR analysis revealed an insertion rate of 91.7% for the VHH genes (**Supplementary Figure S2**). Subsequently, 12 positive clones were sequenced, and their sequences were aligned, demonstrating the high diversity of the library (**Figure 1D**).

### 3.3 Screening and identification of CDV H-specific nanobodies

**Figure 3:**
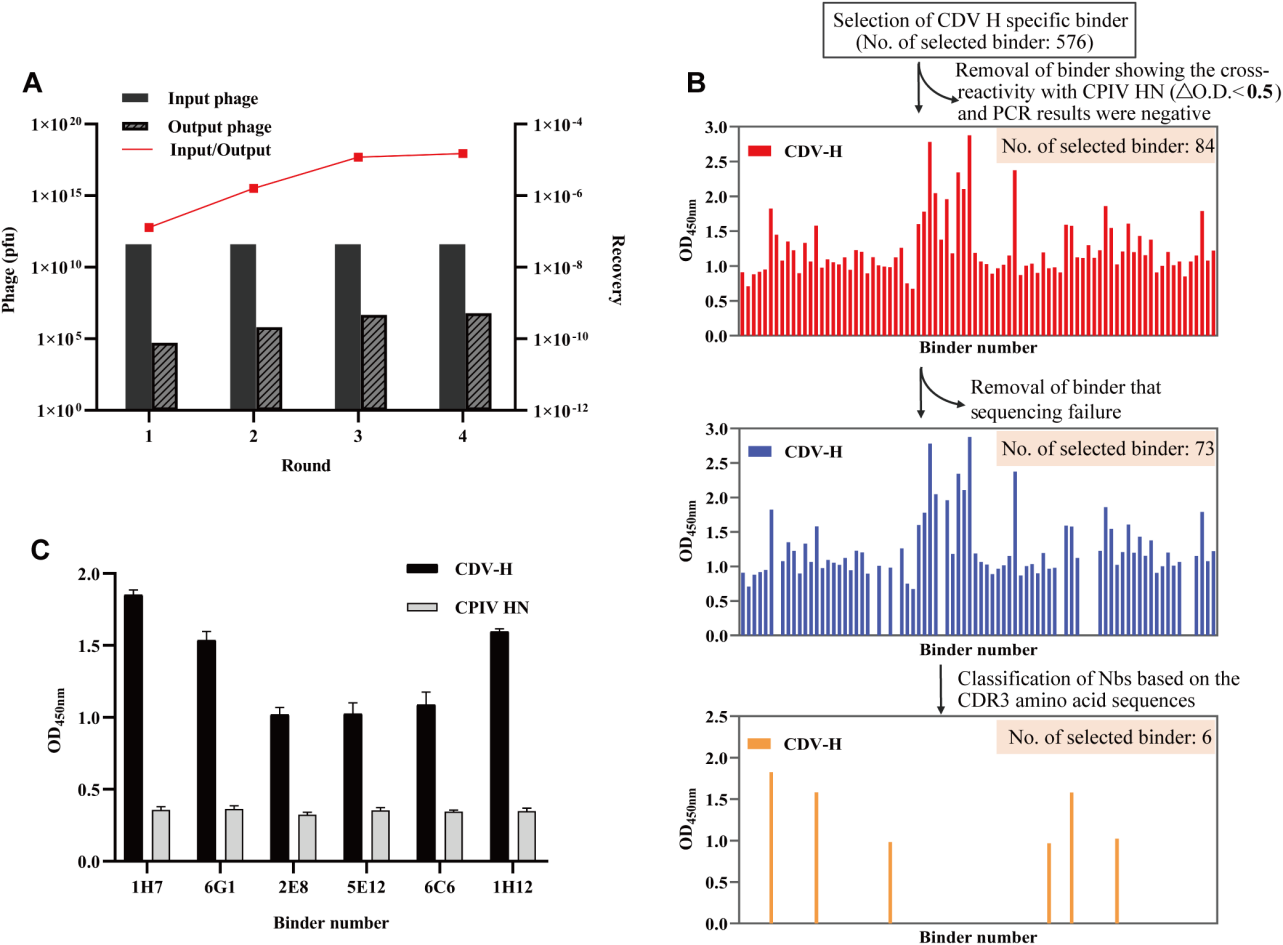
Screening of specific nanobodies against the CDV H protein. **(A)** The anti-CDV H nanobodies were enriched 115.4-fold after 4 rounds of screening. **(B)** Identification of the periplasmic extract of 576 clones to specifically bind to the CDV H protein with indirect ELISA. After sequencing analysis, 6 specific binders were ultimately isolated. **(C)** Specific reactions between the 6 screened nanobodies and CDV H protein.

To screen for nanobodies specifically interacting with CDV H, we utilized the phage antibody library constructed in the previous step for screening. After four rounds of screening, phage clones specific to CDV H were effectively enriched by more than 115.4-fold (**Figure 3A**). Next, 576 phage clones were randomly screened for specific binding to the CDV H protein using ELISA. After nonspecific nanobody binders with high background signals and cross-reactivity to CPIV HN were removed, 84 CDV H-specific clones were isolated (**Figure 3B**). We successfully sequenced 73 positive clones (**Figure 3B**). Subsequently, sequence analysis of the amino acids in the CDR3 region revealed that 6 nanobodies (1H7, 6G1, 2E8, 5E12, 6C6, and 1H12) were obtained. Indirect ELISA results for specific binding indicated that all 6 nanobodies could react with CDV H but not with the His tag control protein CPIV HN, excluding the possibility that the nanobodies recognized the 6xHis region (**Figure 3C**).

### 3.4 Expression of CDV H-nanobodies by *E. coli*

**Figure 4:**
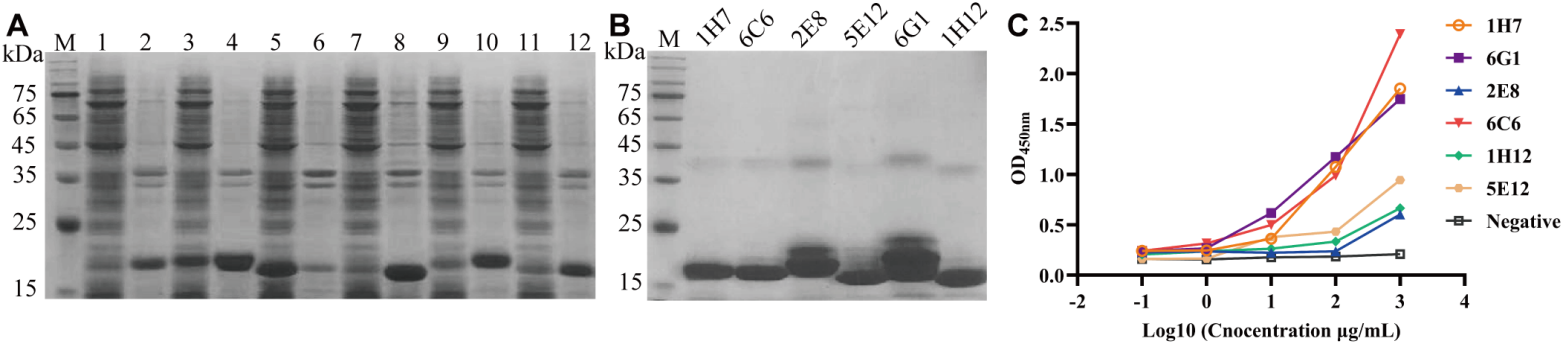
Expression, purification, and identification of the 6 recombinant nanobodies against CDV H protein by prokaryotic system expression. **(A)** Analysis of the expression of the 6 recombinant nanobodies by SDS-PAGE. M: M5 Prestained Protein Marker; lanes 1, 3, 5, 7, 9, and 11: supernatant of Nb-1H7, Nb-6G1, Nb-5E12, Nb-1H12, Nb-2E8, and Nb-6C6 after sonication, respectively; lanes 2, 4, 6, 8, 10, and 12: precipitation of Nb-1H7, Nb-6G1, Nb-5E12, Nb-1H12, Nb-2E8, and Nb-6C6, respectively. **(B)** Analysis of 6 purified recombinant nanobodies by SDS-PAGE. **(C)** Detection of the 6 recombinant nanobodies specifically binding to the CDV H protein using indirect ELISA.

Six VHH genes encoding 1H7, 6G1, 5E12, 1H12, 2E8, and 6C6 were successfully ligated into the pET-28a vectors and transfected into *E. coli* BL21 (DE3) cell lines to assess their expression. SDS-PAGE analysis indicated that Nb-1H7, Nb-6G1, Nb-5E12, Nb-1H12, Nb-2E8, and Nb-6C6 were successfully expressed, with an expected size of 15 kDa (**Figure 4A**). After purification, highly pure Nb-1H7, Nb-6C6, Nb-2E8, Nb-5E12, Nb-6G1, and Nb-1H12 proteins were obtained (**Figure 4B**). Indirect ELISA results revealed that the 6 expressed nanobodies could still bind specifically to CDV H (**Figure 4C**). Among them, 3 nanobodies, Nb-1H7, Nb-6G1, and Nb-6C6, exhibited strong binding activity to CDV H and could be used for further research.

### 3.5 Expression of CDV H-nanobodies in HEK293T cells

**Figure 5:**
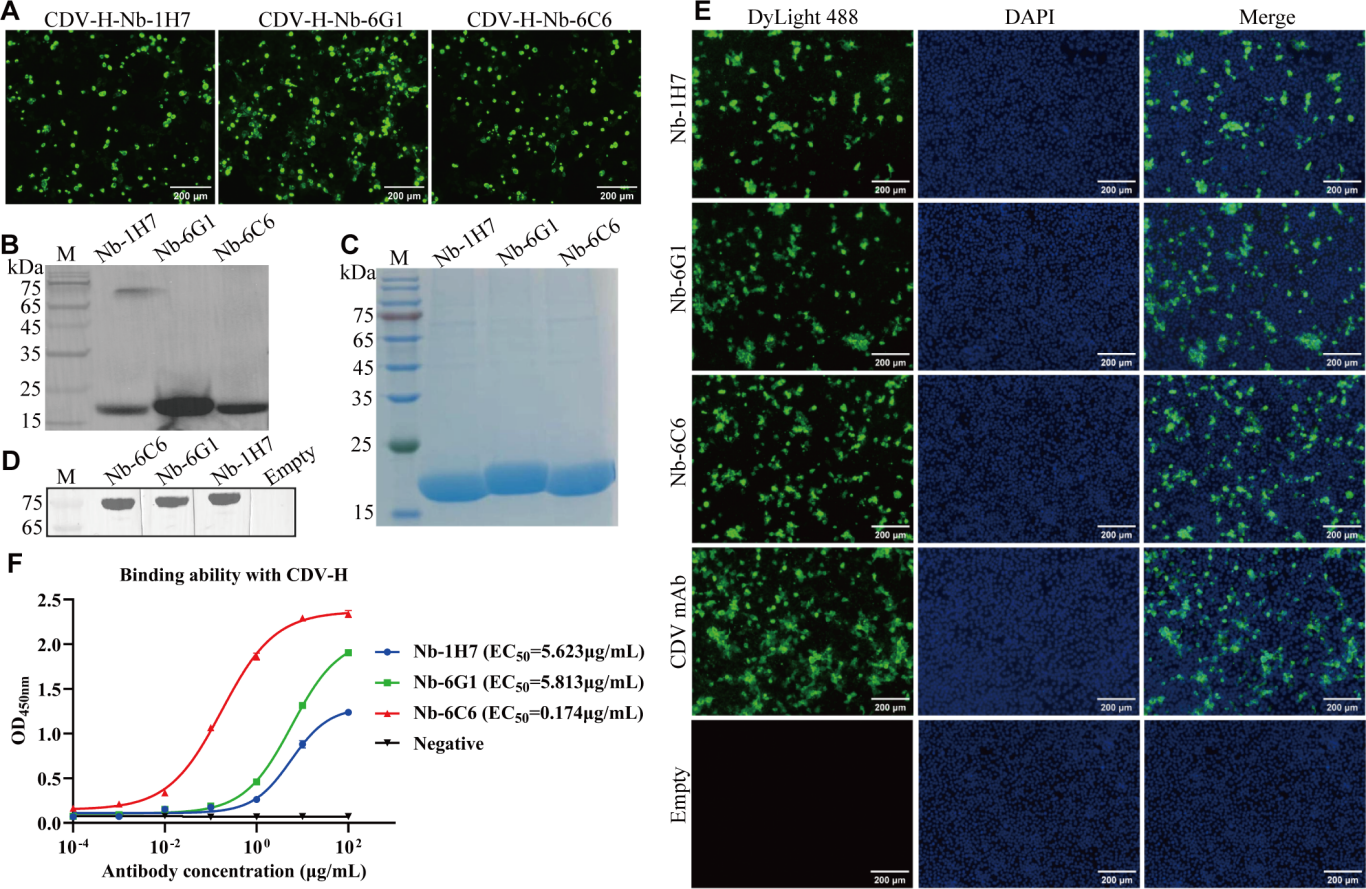
Expression and identification of the three CDV H-Nbs in the HEK293T cells. **(A)** Identification of CDV H-Nbs expressed in the HEK293T cells by IFA. **(B)** Detection of the CDV H-Nbs secreted into the culture medium of HEK293T cells by western blot. **(C)** SDS-PAGE analysis of purified nanobodies Nb-1H7, Nb-6G1, and Nb-6C6. **(D)** Identification of CDV H-Nbs specifically bind to CDV H protein by western blot. **(E)** Detection of CDV in the Vero E6 cells with the 3 CDV H-Nbs by IFA. The monoclonal antibody against CDV was used as a positive control; the supernatant of HEK293T cells transfected with blank vector was the negative control. **(F)** The affinity of Nb-1H7, Nb-6G1, and Nb-6C6 to CDV H at various concentrations. The results are presented as mean absorbance values at OD_450nm_ ± SD (n = 3).

To obtain nanobodies with superior protein conformation, nanobodies were expressed in HEK293T cells using a commercial pcDNA3.4 vector. The IFA results revealed that 3 nanobodies (Nb-6G1, Nb-6C6, and Nb-1H7) were successfully expressed in HEK293T cells using anti-Flag mAb for detection (**Figure 5A**). Western blot analysis revealed that all the proteins were secreted into the medium (**Figure 5B**). SDS-PAGE analysis revealed that the purified Nb-6G1, Nb-6C6, and Nb-1H7 had the expected apparent molecular weights (**Figure 5C**). Additionally, western blot analysis revealed that the 3 purified nanobodies could bind specifically to CDV H (**Figure 5D**). The IFA results further indicated that the 3 nanobodies could detect CDV in Vero E6 cells (**Figure 5E**). Indirect ELISA also revealed that these 3 nanobodies could bind to CDV H, among which Nb-6C6 had the highest affinity (**Figure 5F**), with a half-maximal effective concentration (EC_50_) of 0.174 µg/mL.

### 3.6 Neutralizing activity of CDV H-nanobodies against CDV

**Figure 6:**
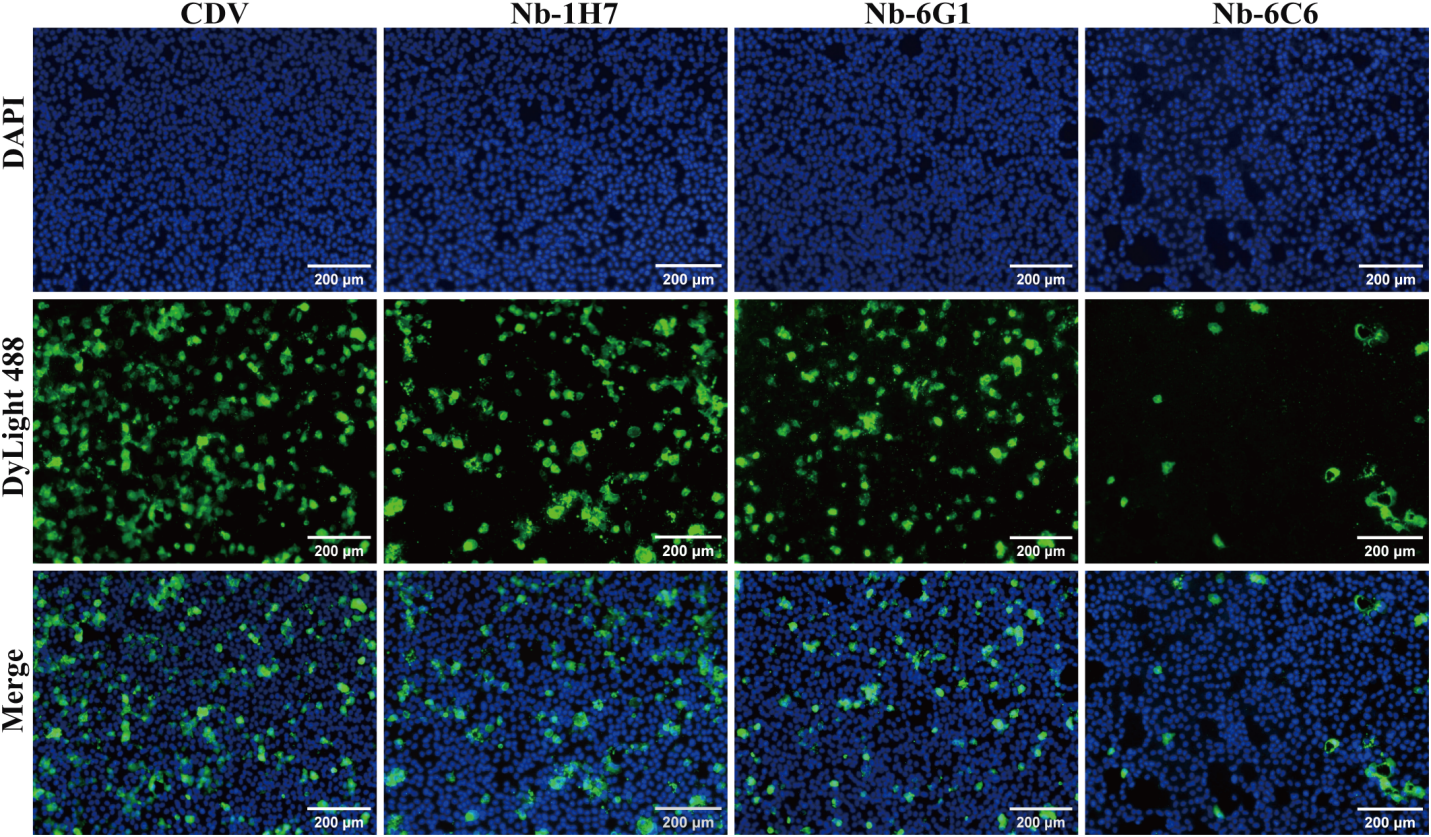
Identification of CDV-neutralizing activity of the nanobodies with specific binding to CDV by IFA.

**Figure 7:**
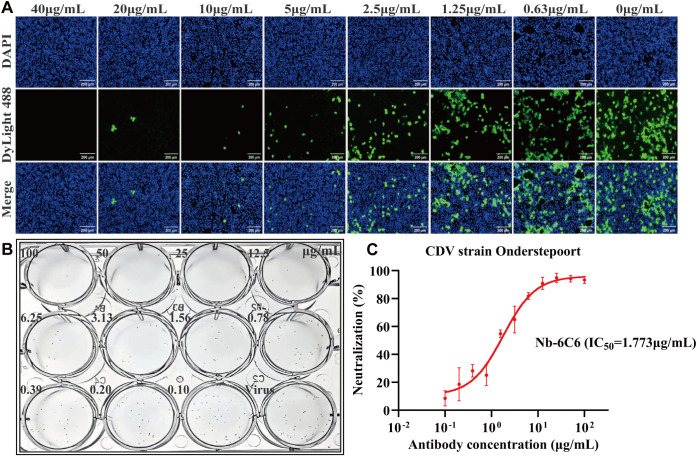
Nb-6C6 neutralization of CDV *in vitro*. **(A)** Green fluorescence images were captured to visualize the gradient-diluted Nb-6C6 neutralizing activity to CDV. **(B, C)** For the calculation of the IC_50_, the foci were counted using the FRNT. The data shown are the mean values for triplicate experiments.

We assessed the neutralizing capacity of nanobodies against CDV infection. The IFA results revealed that among the nanobodies exhibiting specific binding to CDV, Nb-6C6 displayed significant neutralizing activity against CDV (**Figure 6**). These findings suggested that Nb-6C6 was an ideal nanobody for further research. Subsequently, the IFA results suggested that Nb-6C6 could effectively block the entry of CDV into Vero E6 cells in a dose-dependent manner (**Figure 7A**). Additionally, we evaluated the neutralizing activity of Nb-6C6 against CDV using the FRNT. As shown in **Figures 7B, C**, Nb-6C6 potently neutralized CDV in Vero E6 cells, with an IC_50_ value of 1.773 µg/mL.

### 3.7 Construction and characterization of 6C6-Fc

**Figure 8:**
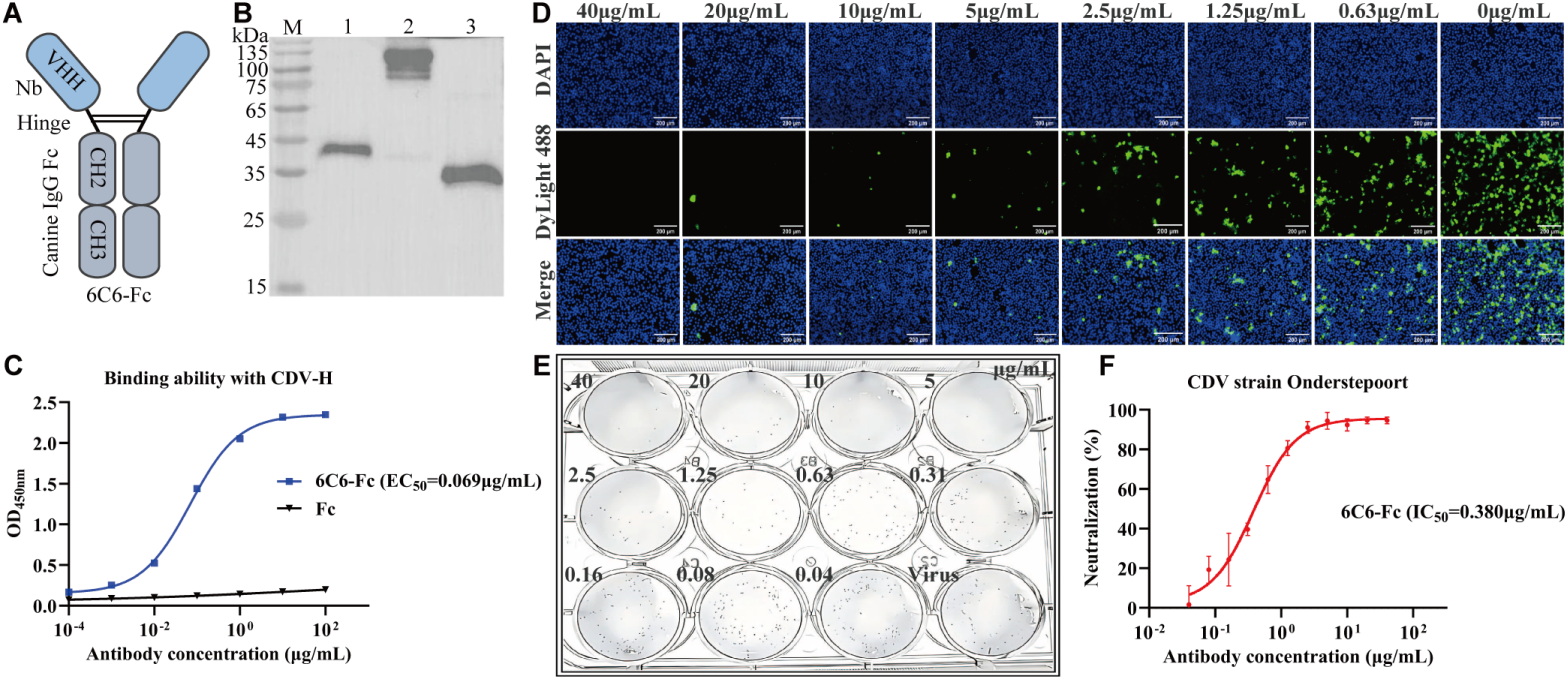
Production and characterization of 6C6-Fc. **(A)** Diagram of the Nb-6C6 fused with canine IgG Fc fragments. **(B)** Analysis of the 6C6-Fc by western blot. M: maker; lane 1: analysis of reduced 6C6-Fc; lane 2: analysis of non-reduced 6C6-Fc; lane 3: expressed Fc protein. **(C)** The affinity of 6C6-Fc to CDV H at various concentrations. The results are presented as mean absorbance values at OD_450nm_ ± SD (n=3). **(D)** Green fluorescence images were captured to visualize the gradient-diluted 6C6-Fc neutralizing activity to CDV. **(E, F)** For the calculation of the IC_50_, the foci were counted using the FRNT. The data shown are the mean values for triplicate experiments.

To enhance Nb-6C6 neutralization capacity, we engineered a canine heavy chain antibody by fusing the canine IgG Fc region to the C-terminus of Nb-6C6 (**Figure 8A**). The resulting 6C6-Fc fusion protein was successfully expressed and secreted in mammalian cell culture supernatants. The constructed 6C6-Fc was approximately 40 kDa under reducing conditions, whereas the nonreduced intact 6C6-Fc (without β-mercaptoethanol) was twice as large (**Figure 8B**). Furthermore, we used the indirect ELISA to evaluate the binding of 6C6-Fc to CDV H. 6C6-Fc specifically reacted with CDV H, whereas the Fc control did not (**Figure 8C**). We found that the binding activity of 6C6-Fc was greater than that of its monovalent form, with an EC_50_ of 0.069 µg/mL. IFA and FRNT revealed that 6C6-Fc neutralized CDV with an IC_50_ of approximately 0.380 µg/mL (**Figures 8D–F**). Compared with the monovalent form of Nb-6C6, the divalent 6C6-Fc exhibited a 4.6-fold increase in neutralizing activity.

### 3.8 Structural basis for neutralization mediated by Nb-6C6

**Figure 9:**
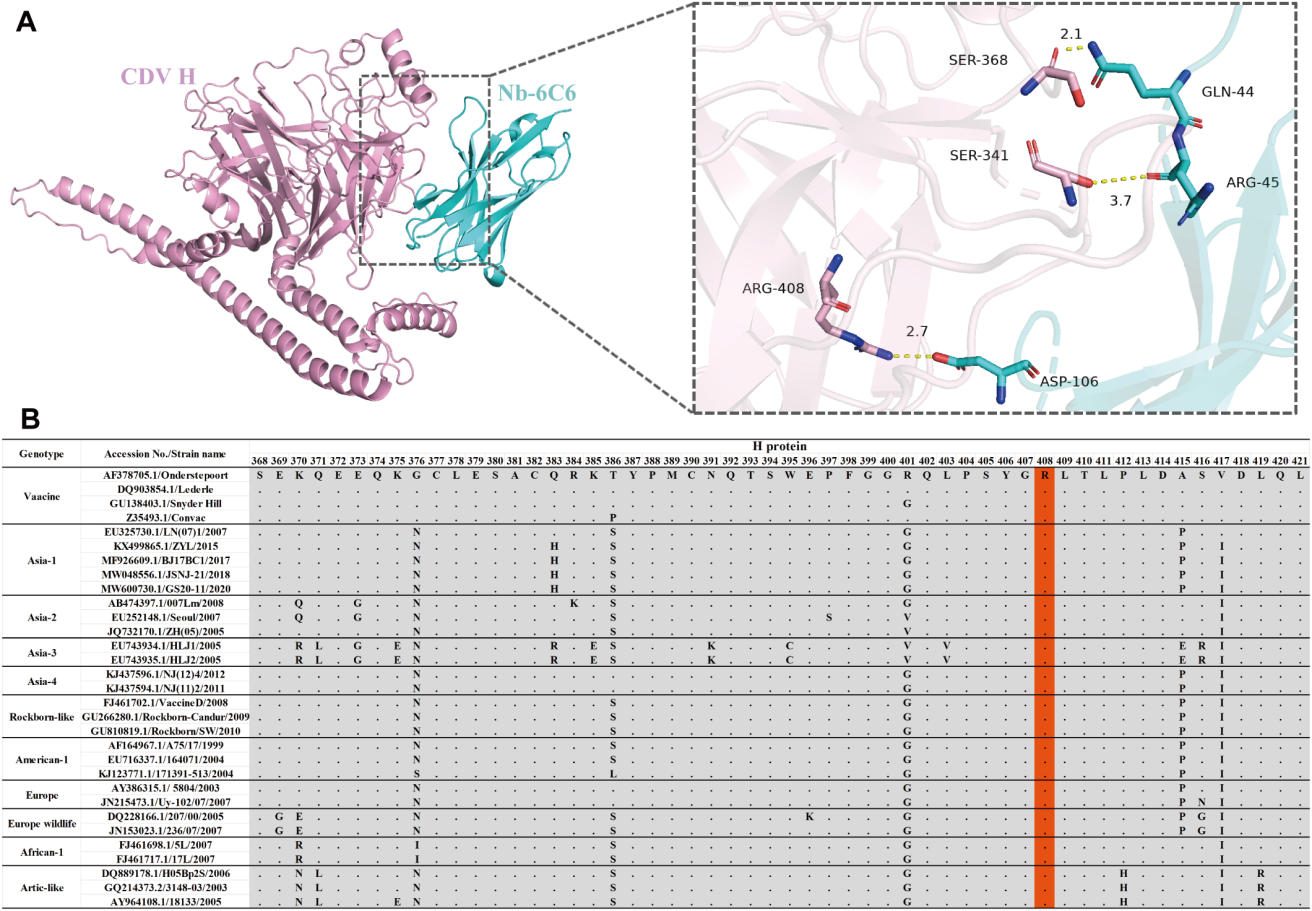
Structural basis of Nb-6C6 neutralization. **(A)** Predicted amino acids are involved in the binding interactions between CDV H (pink) and Nb-6C6 (cyan). The key residues are shown as sticks and H-bonds are shown as yellow dashed lines. **(B)** Amino acid sequence alignment of different CDV H The conserved residue R408 was highlighted with a light red color.

To explore the interactions between Nb-6C6 and the H protein, the AlphaFold3 tool was employed for 3D structure modeling and molecular docking. Through molecular docking, we found that CDV H and Nb-6C6 exhibit good binding energy, with an interface area of 722.7 Å². The docking score was −5.1 kcal/mol, where a smaller value indicates stronger binding energy. Residue R408 of the CDV H was found to form a salt bridge with D106 in the CDR3 region of Nb-6C6. Additionally, residues S368, S341, and R408 of the CDV H could form three hydrogen bonds with residues Q44, R45, and D106 of Nb-6C6, respectively. The hydrogen bond distances were measured as 2.1 Å, 3.7 Å, and 2.7 Å, respectively (**Figure 9A**). Residue R408 of the CDV H is likely a key amino acid for its interaction with Nb-6C6. Amino acid sequence alignment revealed that R408 is highly conserved across different CDV H proteins (**Figure 9B**, light red color).

## 4 Discussion

To date, vaccination combined with drug therapy remains the primary strategy for preventing and controlling CD (41). However, the effectiveness of vaccines is often compromised by the continuous mutation of the CDV, the emergence of new genotypes, and changes in the ecological environment. Although heterologous mAbs have been employed for therapeutic purposes in treating diseased dogs, their efficacy remains unsatisfactory. Therefore, developing novel antiviral drugs against CDV infection is crucial for effective therapeutic purposes. In this study, we successfully isolated a nanobody, Nb-6C6, which exhibits high-affinity binding to CDV H that was successfully expressed in *E. coli* and has neutralizing activity against CDV *in vitro* as determined by IFA and FRNT. To increase its therapeutic potential further, we engineered a bivalent 6C6-Fc fusion protein by fusing Nb-6C6 with the Fc domain of canine IgG. This modification increased the binding affinity for CDV H and improved its neutralizing activity against CDV. We identified the key amino acid sites through structural prediction and molecular interaction modeling through which Nb-6C6 neutralizes CDV.

The utilization of mouse hybridoma technology to prepare specific mAbs has remained an effective approach for treating CDV infection. Bi et al. developed a mouse monoclonal antibody 2D12 targeting the CDV hemagglutinin protein, which demonstrated potent neutralizing activity against CDV infection (42). However, the repeated use of heterologous antibodies for therapeutic purposes can lead to immunogenicity risks and hypersensitivity reactions. These side effects restrict the therapeutic use of mouse-derived mAbs in treating canine diseases (24). Compared with conventional mAbs, nanobodies generated via phage display technology are more amenable to genetic engineering modifications, significantly reducing the risk of immunogenicity in clinical applications. Extensive research has explored the therapeutic potential of nanobodies against critical animal infectious diseases. For example, Chen et al. identified two neutralizing nanobodies (nb14 and nb53) against swine hepatitis E virus (HEV), with nb14 showing complete protection *in vivo* and potential as an antiviral therapeutic for HEV infection (43). Similarly, Boruah et al. created multimeric nanobodies targeting rabies virus glycoprotein, showing enhanced neutralization *in vitro* and partial protection *in vivo*, highlighting their potential as rabies therapeutics (44). Duan et al. developed a nanobody targeting the nucleocapsid (N) protein of porcine reproductive and respiratory syndrome virus (PRRSV), which inhibited viral replication by blocking the self-interaction of the N protein and demonstrated significant antiviral activity both *in vitro* and *in vivo* (45). Therefore, the development of neutralizing nanobodies against CDV holds significant clinical importance. In this study, we identified six nanobody candidates with unique CDR3 sequences. Comparative analysis of prokaryotic and eukaryotic expression systems revealed superior protein conformation in mammalian-expressed nanobodies. Among these, Nb-6C6 demonstrated the highest CDV H binding affinity (EC_50_ = 0.174 µg/mL) and was selected for further characterization. FRNT analysis confirmed its potent neutralizing activity against CDV (IC_50_ = 1.773 µg/mL), highlighting its potential as a therapeutic agent.

Nanobodies generated through phage display technology can be genetically engineered to fuse with the Fc region of IgG to form a bivalent structure. This bivalent form can reduce heterogeneity, enhance immunological functions, and prolong the half-life of nanobodies *in vivo* (46). Importantly, previous research has indicated that bivalent nanobodies have a better affinity than monovalent ones (47, 48). To enhance the neutralization capacity of Nb-6C6, we constructed a bivalent fusion protein, 6C6-Fc, by linking Nb-6C6 to the hinge region and the CH2 and CH3 constant regions of canine IgG. Western blotting under reducing and nonreducing conditions confirmed the successful expression of 6C6-Fc. The western blot findings were consistent with the speculated structural model, indicating that the interchain disulfide bonds in the hinge region could assemble 6C6-Fc into a dimer. Compared with the monovalent Nb-6C6, 6C6-Fc exhibited increased binding affinity and neutralizing activity in functional assays, validating our design strategy. While our *in vitro* data demonstrate that bivalent binding contributes significantly to neutralization potency, previous studies have suggested that the complete antiviral efficacy of Nb-Fc *in vivo* likely involves complementary Fc-mediated effector mechanisms, including ADCC and ADCP (49). Nb-Fc could achieve optimal neutralization through synergistic bivalent binding and Fc effector functions. Furthermore, although the bivalent 6C6-Fc design may confer extended serum half-life based on structural parallels with established Fc-fusion proteins (50, 51), this requires experimental validation. Comprehensive pharmacokinetic studies in relevant animal models will be necessary to definitively establish its *in vivo* persistence and therapeutic potential.

We modeled the structure of the CDV H-Nb-6C6 complex and predicted the molecular interactions between Nb-6C6 and the CDV H protein. Our docking model revealed that residue D106 in the CDR3 region of Nb-6C6 forms hydrogen bonds and salt bridges with residue R408 of CDV H, indicating that R408 is a critical site for their interaction. Hydrophilic interactions, specifically salt bridges and hydrogen bonds, play a crucial stabilizing role in the binding of these proteins. Sequence alignment demonstrated that R408 is highly conserved across different CDV strains, suggesting that Nb-6C6 may have broad-spectrum neutralizing activity against CDV. Based on previous structural determination studies of the binding of CDV H to receptors SLAM or Nectin-4, this key site is not located within the receptor binding site (13). Thus, we speculate that Nb-6C6 cannot inhibit the binding of H to receptors but instead exerts antiviral effects by interfering with the H–F interaction and inhibiting membrane fusion. However, these predictions require experimental validation to confirm their accuracy.

In summary, our study demonstrated that Nb-6C6 and its bivalent fusion protein, 6C6-Fc, exhibit potent neutralizing activity against CDV *in vitro*, highlighting their potential as therapeutic agents. While this study focused on the CDV Onderstepoort strain for neutralization testing, future studies should validate Nb-6C6/6C6-Fc against prevalent genotypes (e.g., Asia-1) to confirm broad-spectrum efficacy. The absolute conservation of residue R408 across CDV genotypes strongly suggests cross-neutralizing potential. Additionally, further studies are needed *in vivo* to evaluate their efficacy and safety in animal models. The findings presented here emphasize the promise of nanobody-based therapies for combating CDV infections in veterinary medicine.

## Data Availability

The original contributions presented in the study are included in the article/**Supplementary Material**. Further inquiries can be directed to the corresponding authors.
