# Supplementary material for: Development of a potent neutralizing nanobody against canine distemper virus hemagglutinin protein
Source: Front Immunol. 2025 May 8;16:1585793. doi: 10.3389/fimmu.2025.1585793 (PMC12095313; doi:10.3389/fimmu.2025.1585793)
Supplement: Supplementary file 1 [file Table1.docx]

**Table S1.** Primers used for this work. The underlying sequences in the primers were restriction sites.

| **Names** | **Sequences (5’-3’)** |
| --- | --- |
| CALL001 | GTCCTGGCTGCTCTTCTACAAGG |
| CALL002 | GGTACGTGCTGTTGAACTGTTCC |
| AlpVHH-F (*Xba*I ) | CAAATGCCTATGCATCTAGACAGKTGCAGCTCGTGGAGTCNGGNGG |
| AlpVHH-R1 (*Eco*RI) | CAACTTTCAACAGTGAATTCGGGGTCTTCGCTGTGGTGCG |
| AlpVHH-R2 (*Eco*RI) | CAACTTTCAACAGTGAATTCTTGTGGTTTTGGTGTCTTGGG |
| Short-F | CTACAAATGCCTATGCATCT |
| Short-R | AACAACTTTCAACAGTGAAT |
| 28a-Nbs-F (*Bam*HI) | CAGCAAATGGGTCGCGGATCCCAGKTGCAGCTCGTGGAGTC |
| 28a-Nbs-R (*Xho*I) | GTCGTCCTTATAATCCTCGAGTGAGGAGACGGTGACCTGGG |
| pcDNA3.4-Nbs-F | TCCTGACTGGGGTGAGGGCCCAGKTGCAGTTGGTTGAGAGC |
| pcDNA3.4-Nbs-R | TCGTCGTCGTCCTTATAATCGGAGCTTACGGTAACCTGTGTGC |
| pcDNA3.4-F1 | GATTATAAGGACGACGACGATAAGG |
| pcDNA3.4-R1 | GGCCCTCACCCCAGTCAG |
| pcDNA3.4-6C6-Fc-F1 | TCCTGACTGGGGTGAGGGCCCAGTTGCAGTTGGTTGAGAGCG |
| pcDNA3.4-6C6-Fc-R1 | CGTTAAACACGGAGCTTACGGTAACCTGTGTGC |
| pcDNA3.4-6C6-Fc-F2 | CGTAAGCTCCGTGTTTAACGAATGCAGATGCAC |
| pcDNA3.4-6C6-Fc-R2 | GATTGTCGACTCTAGAGTCACTTACCAGGTGAGTGGCTCAGG |
| pcDNA3.4-F2 | TGACTCTAGAGTCGACAATCAACCTC |
| pcDNA3.4-R2 | GGCCCTCACCCCAGTCAG |


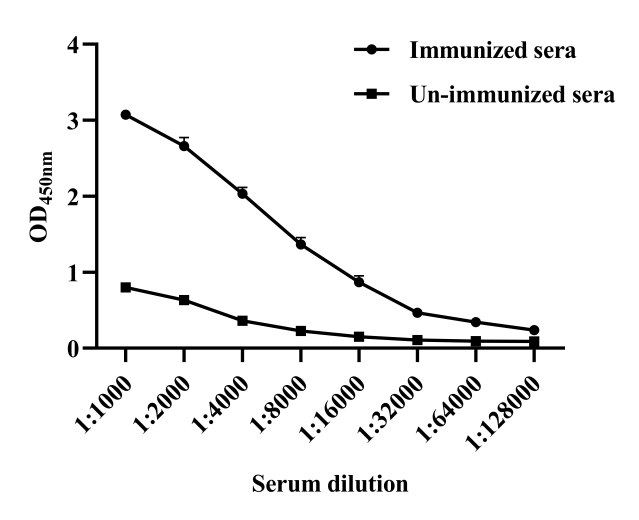


**Figure S1.** Measurement of the titer of anti-CDV H protein antibodies in the serum of immunized alpacas using ELISA.


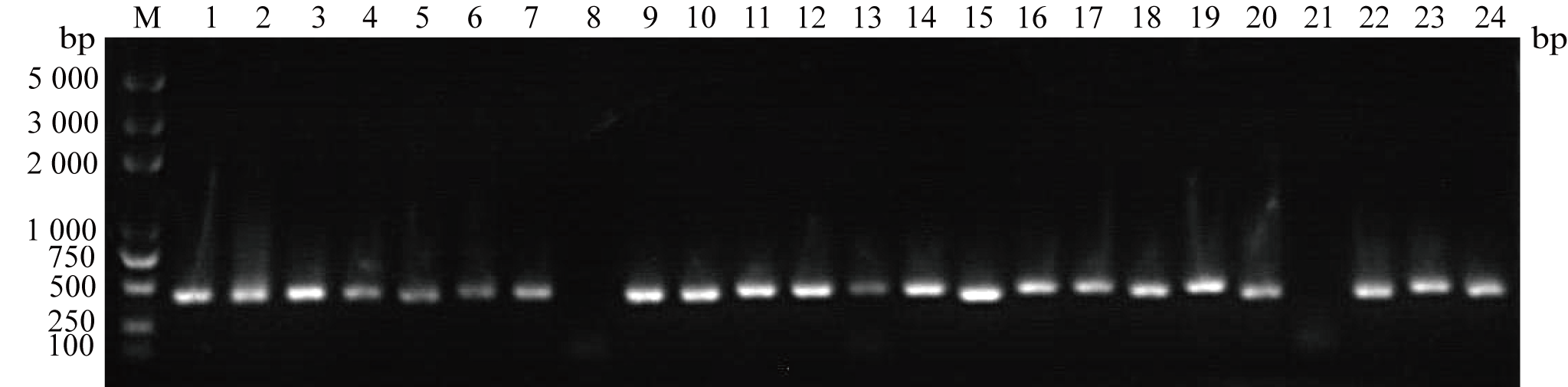


**Figure S2.** Evaluation the insertion rate of correct VHH clones in library. The PCR products of positive clones inserted VHH genes were about 400 bp.
